# Data analysis in medical research: from foe to friend

**DOI:** 10.3325/cmj.2019.60.1

**Published:** 2019-02

**Authors:** Branimir K. Hackenberger

Whether we like it or not, the development of medical knowledge is no longer possible without computers. Sophisticated computerized robotics allows surgeons to operate on patients without even being in the same room with them, while the use of computers by physicians for real-time consultations and second opinions is not rare anymore. While in some cases the role of computers is not very obvious, in others medical devices are almost inseparable from the computer. A good example of the latter would be computer tomographs, positron emission tomographs, and magnetic resonance scanners.

Apart from powerful hardware, computer functioning requires sophisticated software, whose development is based on superior and functional quantitative methods and depends on the progress of mathematics and data science. Furthermore, to accelerate the development of mathematics and data science, it is particularly important to have data input and articulation of problems from medicine. Despite the need for the development of medical informatics technology, we are frequently faced by a strong resistance toward quantitative methods. In scientific work, this resistance is especially directed toward statistical methods.

Why do so many people hate statistics? Do we need statistics anyway? Why? Unfortunately, there is much animosity toward statistics and it is often considered to be the necessary evil. The main reason lies in the animosity toward mathematics and in the belief that only mathematicians can understand statistics. The premise that statistics and mathematics are too tricky to understand keeps many people from further attempts at understanding. However, statistics represents something entirely different for researchers than for statisticians or mathematicians, in the same way as the microscope represents something different for physicists or mechanical engineers than for microbiologists or technicians.

For researchers, statistics is just a tool, but a powerful tool that allows taking a step forward in scientific research. Statistical methods are essential for the inference of argument and decision-making and are central to proving that the measured quantity is the result of natural laws rather than of coincidence. Apart from well-known and widely used classical statistics, eg, frequentist statistics, there are many more modern, sophisticated statistical methods that make research even more challenging. Perhaps the most famous is Bayesian statistics – a very powerful but still insufficiently used method in informatics technology.

The resistance toward statistics in medical community, but also the potential enormous applicability of these methods, prompted me to write a series of texts that will be published as a column in the *Croatian Medical Journal*. The series will deal with the possibilities of introducing new experimental designs in laboratory and clinical research, as well as the implementation of machine learning, deep learning, and big data in medicine in a simplified and easily understandable manner, with a hope of dispelling the myth of statistics as being too complicated to be comprehended by an average medical researcher.

